# On the central role of brain connectivity in neurodegenerative disease progression

**DOI:** 10.3389/fnagi.2015.00090

**Published:** 2015-05-21

**Authors:** Yasser Iturria-Medina, Alan C. Evans

**Affiliations:** ^1^Montreal Neurological InstituteMontreal, QC, Canada; ^2^Ludmer Center for NeuroInformatics and Mental HealthMontreal, QC, Canada

**Keywords:** brain connectivity, deregulated gene networks, misfolded proteins, neuronal activity toxicity, metabolic dysfunction, vascular deregulation, disease spreading, neurodegeneration

## Abstract

Increased brain connectivity, in all its variants, is often considered an evolutionary advantage by mediating complex sensorimotor function and higher cognitive faculties. Interaction among components at all spatial scales, including genes, proteins, neurons, local neuronal circuits and macroscopic brain regions, are indispensable for such vital functions. However, a growing body of evidence suggests that, from the microscopic to the macroscopic levels, such connections might also be a conduit for in intra-brain disease spreading. For instance, cell-to-cell misfolded proteins (MP) transmission and neuronal toxicity are prominent connectivity-mediated factors in aging and neurodegeneration. This article offers an overview of connectivity dysfunctions associated with neurodegeneration, with a specific focus on how these may be central to both normal aging and the neuropathologic degenerative progression.

## Introduction

Intra-brain connectivity is indispensable for the attainment and maintenance of animal life. Genes, proteins, neurons, cell assemblies and gross brain regions all interact constantly to orchestrate the brain functions that underly sensorimotor and cognitive processing. Homeostatic mechanisms establish a basal level of functional organization upon which subtle variations are overlaid that subserve the changes in mood, attention, performance and response to external stimuli that we discern at the behavioral level. However, this delicate equilibrium may break down, particularly in the presence of neurological and psychiatric disorders where aberrant pathologic factors provoke massive alterations in connectivity at all brain levels (Konrad and Eickhoff, 2010; Bicchi et al., 2013; Iturria-Medina, 2013; Reynolds and Stewart, 2013; Gomez-Ramirez and Wu, 2014; He and Evans, 2014; Pievani et al., 2014). Recent advances in brain mapping tools, including genetics, electrophysiology and imaging techniques, with the support of new bioinformatic analysis, have extended to unprecedented levels our understanding of segregation and integration processes in the normal brain (Stam and van Dijk, 2002; Bota et al., 2003; Hagmann et al., 2007; Iturria-Medina et al., 2007; Karlebach and Shamir, 2008; Axer et al., 2011; Friston, 2011; Sporns, 2011; Evans, 2013). Additionally, they have revealed the connectional alterations associated with a wide range of psychiatric and neurological disorders (Buckholtz and Meyer-Lindenberg, 2012; Meyer-Lindenberg and Tost, 2012). Both brain disconnections and hyperconnections are commonly observed for different neurodegenerative diseases (for detailed review see Pievani et al., 2014). How disconnections and hyperconnections arise and coexist during disease progression is still not well understood. Often, disconnections are considered a direct consequence of neurodegeneration, while hyperconnections are assumed to reflect compensatory mechanisms or spatiotemporal correlation in pathology. But such views may be a simplistic interpretation of more complex phenomena, in which brain connectivity could be playing a more causal role. In this article, we provide a brief overview of the biological mechanisms implicated in connectional dysfunctions and consequent neurodegeneration. The article is organized in four primary subsections. The first offers a brief overview of gene regulatory network alterations and their role in neurodegeneration. The second reviews the demonstrated role of the brain’s structural architecture on prion-like propagation, as a main factor mediating neurodegenerative progression. The third presents and discusses the evidence supporting the neuronal activity dependent neurodegeneration hypothesis, in which functional connectivity presents an active role. The fourth integrates previous and recent findings, emphasizing the role of multimodal connections on disease spreading and progression. Finally, we highlight some outstanding questions and the challenges in building an operational model of dynamic brain organization that can account for both normal brain aging and neurodegenerative disease.

## Alterations in Normal Brain Connectivity and Neurodegeneration

### Deregulated Gene and Protein Networks

**Figure 1:**
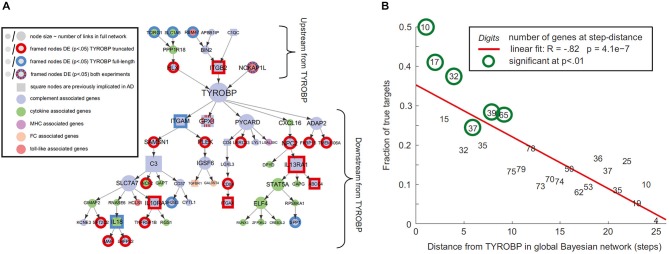
**Connectivity distances to pathologic epicenters predict diseases effects. (A)** TYROBP causal network in late onset Alzheimer’s disease (AD), **(B)** differential expression levels of deregulated genes associated with TYROBP at various functional distances from it. Note the negative association (*R* = −0.82; *P* < 10^−3^) implying increasing impact with proximity to TYROBP. Figure adapted from Zhang et al. (2013), with permission from Elsevier.

Gene regulatory networks control the expression levels of mRNA and proteins. Normal cellular activity depends upon the proper functioning of these networks. This makes the analysis of regulatory network dynamics a crucial step towards understanding the biological processes of health and disease (for reviews, see Bota et al., 2003; Karlebach and Shamir, 2008; Bernot et al., 2013). Aging and neurodegeneration are thought to have strong upstream genetic causes. For instance, Apoeε4 and BCHE genes are considered important risk factors for the development of Alzheimer’s disease (AD; Genin et al., 2011; Cramer et al., 2012; Ramanan et al., 2014). Meanwhile, increasing evidence supports the important modulatory impact of many other AD-related genes (Lambert et al., 2013). Similarly, amyotrophic lateral sclerosis (ALS) is associated with different genetic risk factors, e.g., TDP-43 and SOD, which act in combination with aging and environmental conditions (for reviews see Al-Chalabi and Hardiman, 2013; Robberecht and Philips, 2013). Such multi-factorial causes during the neuropathologic progression are common for the most prevalent neurodegenerative diseases of AD, ALS, Frontotemporal dementia (FTD), Parkinson’s disease (PD) and Huntington’s disease (HD). It strongly supports that “aberrant” genes do not act alone on pathologic progression but by their interaction with other collaborator genes under the influence of environmental/experience conditions (e.g., life style, epigenetic effects). Nutrition conditions have an important modulatory role on gene activities and aging disorders (Joseph et al., 2009; Bouchard-Mercier et al., 2013; Nicolia et al., 2014). For instance, caloric restriction and diet rich in anti-inflammatory and antioxidant properties have been found associated to increased longevity and preserved cognitive functioning (Roth et al., 2002; Colman et al., 2009; Joseph et al., 2009; Stice et al., 2013; Crichton et al., 2013; Sezgin and Dincer, 2014). *Nutri-epigenomics* science focus on the influence of nutrition on epigenetic modifications and its consequences on health (Gallou-Kabani et al., 2007), which ideally should contribute to develop effective nutrition-based therapeutic interventions. Modern gene expression profiling techniques allow us to quantify gene-specific activity across different tissues and time points (O’Driscoll, 2011). As a result, genetic interaction occurring between regions of interest can be characterized by means of sophisticated statistical concepts and tools (Bota et al., 2003; Karlebach and Shamir, 2008; Bernot et al., 2013), thereby contributing to our understanding of how regulatory networks are involved in disease progression (Crespo et al., 2012; Narayanan et al., 2014; Leiserson et al., 2015). In the context of brain degeneration, Zhang et al., 2013, reported a remarkable example of causal gene-gene pathologic interaction. These authors used gene expression profiles of the prefrontal cortex to identify regulatory networks causally associated with late onset AD. They identified an immune and microglia-specific gene module that is strongly regulated by the gene TYROBP, directly associated to Amyloid-β (Aβ) turnover and neuronal damage. This TYROBP causal network (Figure 1A), characterized in detail by means of Bayesian network analysis, showed a direct modulatory effect on late onset AD gene networks, which was verified not only in human brain but also in an experimental animal model. A salient finding was that the differential gene expression observed for late onset AD presented a distance-dependent relationship with TYROBP (Figure 1B). Those genes with a higher functional association with TYROBP are more likely to be altered during the disease process, as well as to propagate the pathologic effects to their connected neighbors. Although this characteristic gene network regulatory effect (Zhang et al., 2013) needs further exploration and validation in other neurodegenerative diseases, it illustrates how connectional links at the molecular level can mediate disease propagation.

### Inter-Cellular Misfolded Proteins Propagation Across Structural Pathways

**Figure 2:**
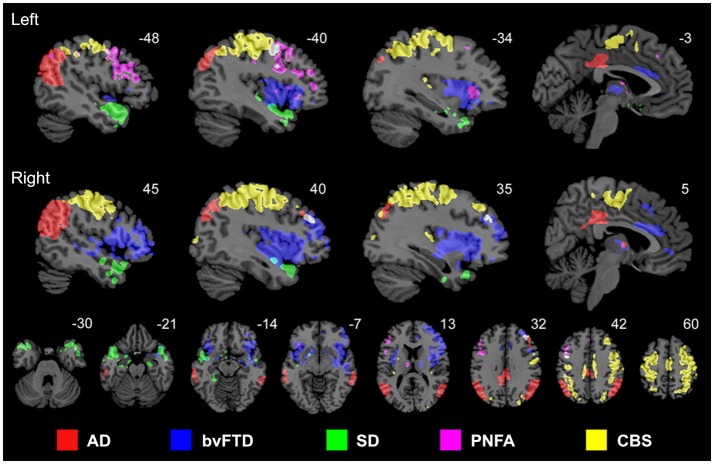
**Anatomically dissociable networks targeted by five different neurodegenerative disorders: AD, behavioral variant frontotemporal dementia (bvFTD), semantic dementia (SD), progressive nonfluent aphasia (PNFA), and corticobasal syndrome (CBS)**. Figure adapted from Seeley et al. (2009), with permission from Elsevier.

Proteins that fail to configure properly are called misfolded proteins (MP). Historically, they have been causally associated with aging and several human neurodegenerative diseases (Braak and Braak, 1991; Dobson, 2002, 2003; Braak et al., 2004). The prion-like hypothesis proposes that cell-to-cell transmission of toxic MP is a principal cause of neurodegeneration (Frost et al., 2009; Brundin et al., 2010; Hallbeck et al., 2013). Increasing neuropathologic evidence supports the spread of MPs from initial host regions to anatomically connected areas, spreading and simultaneously re-seeding the toxic effects (Frost et al., 2009; Waters, 2010; Nath et al., 2012; Jucker and Walker, 2013; Song et al., 2014). This fact, combined with recent evidence supporting the notion that each neurodegenerative disorder is associated with a characteristic group of MPs (Brundin et al., 2010), motivated in part the network degeneration hypothesis (NDH; Seeley et al., 2009). This hypothesis proposes that each disorder should present disease-specific anatomic, functional and metabolic pathways. Seeley and colleagues used MRI to demonstrate that different neurodegenerative disorders are associated with spatially dissociable atrophy patterns, each pattern corresponding to a consistent structural covariance and functional sub-network (Figure 2; Seeley et al., 2009). In a complementary study (Zhou et al., 2012), the same group showed that regions with higher connectivity with, and shorter functional distances to, disease-specific epicenters presented greater structural atrophy. Raj et al., 2012, introduced a diffusion network model of intra-brain MP propagation, according to which the increase over time of the number of diseased afferents from a given brain region to any other region depends upon the disease concentration factor in both regions and upon the anatomical connection strength between them (Raj et al., 2012). From this model, an analytical expression for structural atrophy dynamics was obtained. After a mathematical decomposition of a healthy brain anatomical connectome, the authors found a significant correspondence between specific dissociable connectivity modules and the characteristic atrophy patterns of different neurodegenerative diseases (AD, behavioral variant FTD [bvFTD]). Each connectivity module’s weight in the initial connectome was inversely proportional to the population prevalence of a specific disorder (AD, bvFTD or HD). This suggested that the final structural atrophy pattern in adulthood could be the weighted combination of characteristic atrophy patterns from prevalent neurodegenerative diseases, in which each disease-characteristic pattern is weighted by the individual predisposition to express such disease. In general, these three seminal studies (Seeley et al., 2009; Raj et al., 2012; Zhou et al., 2012) supported the NDH, as well as the structural and functional connectivity-mediated spread of neuropathologic effects. However, neurodegenerative gray matter atrophy patterns may not be uniquely provoked by MP toxicity. Other pathologic factors, such as neuronal activity toxicity, and metabolic and vascular deregulations (see below) may contribute to cell death.

**Figure 3:**
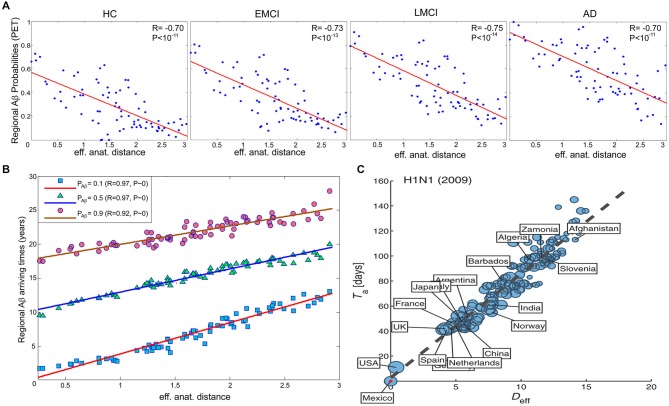
**In brain and social networks, effective proximity to an epicenter modulates the propagation of aberrant factors. (A)** PET-based regional Aβ deposition probabilities for different clinical groups (healthy control (HC), early mild cognitive impairment (EMCI), late mild cognitive impairment (LMCI) and AD) vs. effective anatomical distances to the identified Aβ outbreak region (anterior and posterior cingulate cortices). **(B)** Regional Aβ arriving times vs. effective anatomical distances, for different Aβ probability thresholds (i.e., 0.1, 0.5 and 0.9). **(C)** N1H1 pandemic arrival time vs. effective distance (D*_eff_*) to outbreak country (i.e., Mexico). In **(C)**, the effective distance was computed from the projected global mobility network between countries. Panels **(A,B)** and **(C)** were adapted with permission from Iturria-Medina et al. (2014), and Brockmann and Helbing (2013) respectively.

In order to obtain straight evidence of MP spread as a function of anatomical proximity to a disease propagation epicenter, Iturria-Medina et al. (2014), analyzed PET Aβ deposition patterns in 733 healthy and diseased brains. Motivated by the remarkable similarity between intra-brain pathology propagation and the spread of human infectious diseases in social networks, we hypothesized that MP dynamics can be mathematically described and characterized by the epidemic-like interactions between infection agents (the aberrant proteins) and the brain’s defense response, mediated by the brain’s anatomical architecture (Iturria-Medina et al., 2014). The proposed epidemic spreading model (ESM) reproduced Aβ patterns from healthy to advanced diseases states, allowing the reconstruction of individual lifetime histories of intra-brain Aβ propagation, and the subsequent analysis of the biological factors that promote such propagation/deposition (e.g., the relationship of clinical state with MP production and/or clearance). When exploring the relation between regional Aβ deposition pattern and the connectional proximity to the Aβ outbreak regions, as identified by the ESM (anterior and posterior cingulate cortices), a significant negative linear trend was observed (Figure 3A), with more advanced disease states corresponding to higher deposition. Also, a significant negative relation between regional anatomical connectivity degree and Aβ arrival time (measures of hubness and disease vulnerability) was observed (Figure 3B). This relation was independent of the selection of different Aβ deposition thresholds, indicating that regions with a higher degree of anatomical connectivity experience earlier Aβ arrival and, consequently, larger periods of exposure to the toxic effect of the aberrant protein. Interestingly, and supporting the hypothesis of an epidemic spreading behavior for MP propagation, a similar linear predictive relationship has been reported for effective distance in human social networks and disease arrival times for real epidemic propagation of infectious disease (Brockmann and Helbing, 2013) (e.g., 2009 H1N1 pandemic; see Figure 3C).

Also in line with the prion-like hypothesis, the phosphorylated 43 kDa TAR DNA-binding protein (pTDP-43) has been identified as a major neuropathologic factor in ALS and frontotemporal lobar degeneration (Neumann et al., 2006; Geser et al., 2009). Recently, Brettschneider et al. (2013), identified four characteristic stages of pTDP-43 neuropathology in ALS, which suggested a sequential pTDP-43 intra-brain dissemination pattern (Brettschneider et al., 2013). Schmidt et al. (2015), found a dense level of anatomical connectivity between the regions of these four pTDP-43 stages. These authors also used a computational random walker spread model to simulate axonal spread of the pTDP-43 factor as a walking particle along the white matter pathways (Schmidt et al., 2015). Consistent with the hypothesis that pTDP-43 pathology is propagated along axonal pathways, they observed a significant overlap between the simulated pTDP-43 patterns and the sequential distribution found previously in ALS autopsy cases.

### Neuronal Activity-Dependent Neurodegeneration

The upstream causal role of MP on neurodegenerative disorders is currently under scientific controversy (Soto and Castilla, 2004; Hilker et al., 2011). MP presence do not always correlate well with structural atrophy and/or cognitive decline levels, whereas therapeutic drugs created to reduce MP levels have demonstrated poor modulatory effects on disease progression (Holmes et al., 2008). Different alternative hypotheses have been proposed in order to fit the inconsistencies of the MP prion-like assumptions. For example, the Caspase-6 neurodegeneration hypothesis of AD (LeBlanc et al., 1999; Albrecht et al., 2007; LeBlanc, 2013), explains cell inflammation and death by the stress-associated action of the Caspase-6 enzyme. Caspase-6 activation modulates also Aβ and phosphorylated tau concentrations, which are strongly associated to the stress in neurons and cell lines. Moreover, consistent evidence suggests that abnormal neuronal and synaptic activity may modulate brain MP levels (Kamenetz et al., 2003; Cirrito et al., 2005, 2008; Buckner et al., 2009; Bero et al., 2011). For instance, exogenous increases in neuronal and synaptic activity in the hippocampus, induced by electric stimulation, increase the extracellular Aβ concentrations in that region (Cirrito et al., 2005). Also, endogenous neuronal activity changes have an equivalent impact on Aβ concentrations (Bero et al., 2011), suggesting that regional differences in basal neuronal activity levels could explain regional vulnerabilities to Aβ presence and toxicity. From these facts arise some relevant questions: can aberrant neuronal/synaptic activity have an upstream role in neurodegenerative progression, and, importantly, is functional connectivity a mediator of neuronal/synaptic toxicity spreading? Motivated by these questions, de Haan et al. (2012), used neural mass modeling to explore local neuronal activity in relation to large-scale connectivity in normal and abnormal conditions. For this, the authors simulated neural dynamics using a real structural brain connectome, and induced progressive damage to the regions based on their level of activity. The results suggested that, in no-task conditions, hubs should be the most active regions (due to the convergence of heteromodal activity), and also that excessive connectivity-dependent neuronal activity can have a significant role in the neurodegenerative progression, thus explaining the associated hub vulnerability (de Haan et al., 2012). Previously, a robust relationship between regional hubness (in terms of functional connectivity) and Aβ depositions had been reported (Buckner et al., 2009), whereas functional hyperconnectivity, mainly between cingulate and medio-temporal regions, had been associated with semantic memory deficits (Gardini et al., 2015). Similarly, in AD, regional metabolic alterations had been found to follow Aβ presence in many brain regions (Förster et al., 2012), whereas the spatial mismatch between these two pathologic components can be explained by functional connection to Aβ binding areas (Klupp et al., 2014). This means that non-Aβ areas can also be metabolically deregulated during disease progression if those areas are functionally linked to Aβ and/or functionally-impaired zones. Moreover, derangement of metabolic connectivity patterns have been associated with elevated Aβ burden (Carbonell et al., 2014a,b), while a significant modulatory impact of the Apoeε4 genotype on hypometabolism had been observed (Jagust and Landau, 2012; Carbonell et al., 2014a). All together, these results support the contention that neuronal/synaptic toxicity spreading in neurodegeneration, and associated activity-dependent deregulation of local MP and metabolic levels, strongly depend on anatomic, functional and metabolic brain connectional patterns.

### Towards a Multi-Factorial Disease Spreading Perspective

**Figure 4:**
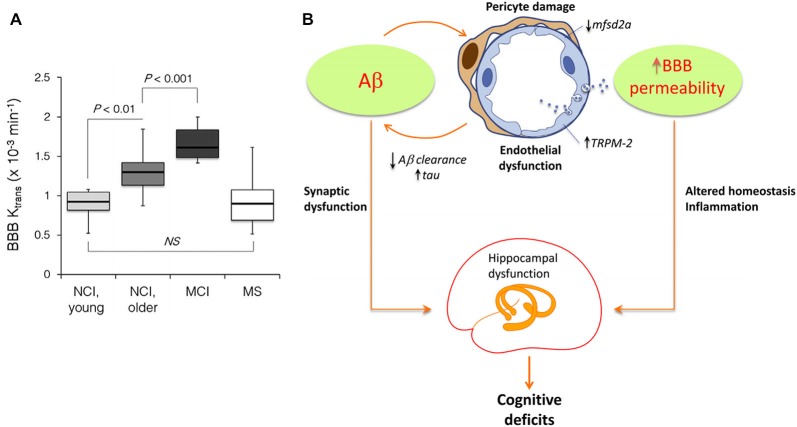
**Age-dependent blood-brain barrier (BBB) permeability breakdown may be causally associated with neurodegenerative spreading (Panels (A) and (B) were adapted from Montagne et al. (2015), Iadecola (2015), respectively, with permission from Elsevier). (A)** Significant increases in BBB permeability in older compared young group of individuals with no cognitive impairment (NCI), and MCI compared to older NCI group in the entire hippocampus. Multiple sclerosis (MS) patients with no cognitive impairment were comparable with the age-matched young NCI group (Montagne et al., 2015). **(B)** Hypothetical pathologic mechanisms by which Aβ may induce BBB permeability alterations and hippocampal/cognitive dysfunction (Iadecola, 2015). Aβ affects endothelial cells, damaging pericytes, vesicular transport and Ca^+^ balance. This contributes to BBB disruption, homeostasis alterations, reduction on misfolded proteins (MP) clearance and tentatively to hippocampal dysfunction, cognitive deficits and intra-brain pathology spreading.

Although generally associated with specific hypotheses, previously proposed pathologic mechanisms are not unrelated. In addition to have a modulatory impact on protein expression, gene activity is markedly associated with structural connectivity patterns (French and Pavlidis, 2011; Wolf et al., 2011a; Ji et al., 2014; Fakhry and Ji, 2015) and synaptic density dynamics (Goyal and Raichle, 2013). This suggests that alterations in gene regulatory networks or aberrant signal spreading across them may induce important changes in structural, functional and metabolic brain patterns, even as an additional downstream effect of a main genetic pathologic factor. Similarly, strong associations persist among different forms of brain connectivity, under normal or abnormal conditions. The vascular and metabolic/functional systems represent a remarkable example. Among other relevant functions, the vascular system supplies oxygen, glucose and other nutrients, and clears away deoxygenated blood and metabolic products (Scremin, 2012). These functions are essential to satisfy daily neuronal/glial energy and maintenance demands. However, this close association dates from initial neurodevelopmental processes: axon-guidance cues mediate the navigation of blood vessels along predestined tracks during development (Carmeliet and Tessier-Lavigne, 2005; Zacchigna et al., 2008), whereas angiogenic vascular endothelial growth factor regulates the migration of various neuron types to their final destination (Schwarz et al., 2004; Zacchigna et al., 2008). Recently, Lacoste et al. (2014), combined genetics, imaging and computational tools to verify that neural activity changes can modulate vascular networks. They found that decrease or enhancement of neural activity (by deafferentation, (de)stimulation or genetic impairment of neurotransmitter release) leads to equivalent effects in vascular density and branching (Lacoste et al., 2014). Together, these facts explain the anatomical positioning and behavioral similarities that have also been uncovered among the vascular and the functional/metabolic pathways (Melie-García et al., 2013; Jann et al., 2015). Moreover, the vascular system plays a major role in aging and associated neurodegenerative processes (Zacchigna et al., 2008; Quaegebeur et al., 2011; Iadecola, 2013). Capillary density loss and other vascular abnormalities have been consistently observed in healthy aging, AD, leukoaraiosis (LA) and HD (Brown and Thore, 2011; Wolf et al., 2011b). Damage to vascular network integrity leads to MP clearance deficits and resultant deposition. For instance, the efflux across the blood-brain barrier (BBB) contributes Aβ clearance (Deane et al., 2009; Qosa et al., 2014). Qosa et al. (2014), reported that around a 60% of soluble Aβ^40^ is cleared across BBB while the remaining is cleared by brain degradation. Consistent with this thesis, a significant age-dependent BBB permeability breakdown, that correlates with cognitive dysfunction, has been observed in human hippocampus (Montagne et al., 2015; see Figure 4A). Such aging effects have a crucial impact on BBB-mediated MP clearance and deposition (Iadecola, 2015; see Figure 4B), contributing to structural, functional and metabolic connectional deregulation in a continuous degenerative cycle. In addition, brain neuroinflammation is characteristic feature during neurodegeneration (Streit et al., 2004; Block et al., 2007; Lull and Block, 2010). It is particularly associated to microglia cells activity, which reacts defensively in respond to different events, such as infection, brain injury or associated autoimmune processes (Gendelman, 2002). Under certain pathologic conditions (ex. presence of environmental toxins or neuronal damage), microglias can enter to a hyperactivation state and release excessive reactive oxygen species (ROS), which cause neurotoxicity and cell death (Block et al., 2007; Lull and Block, 2010). Then, local pathologic effects associated to microglia-mediated neuroinflammatory processes may impact other connected areas. Similarly that with the region-region transmission of previously mentioned aberrant factors (ex. MP, toxic neuronal/synaptic signals, metabolic deregulation, BBB damage), functional/metabolic impairment and neuronal death in a given brain region, due to neuroinflammation and ROS, may alter its vascular, functional, metabolic and anatomical links, and gradually the multi-factorial subnetworks associated to the connected regions, extending the negative neuroinflammatory effects across the interconnected brain.

## Discussion and Conclusions

**Figure 5:**
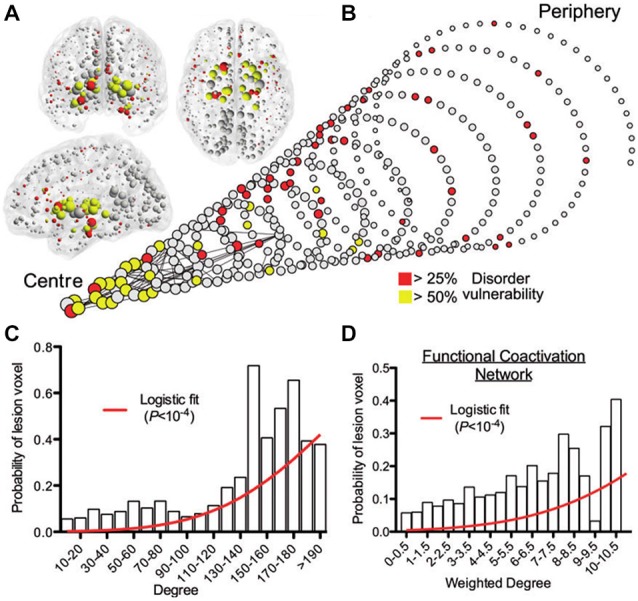
**Gray matter lesions identified on 26 clinical brain disorders impact mainly on the structural and functional hub regions. (A)** Nodes of the normative structural connectome, represented in anatomical space, with nodes size reflecting connectivity degrees. **(B)** Spiral representation of the region vulnerability vs. hubness relationship. Nodes of similar degree are arranged in the same circle, and the different circumferences arranged so that the tip of the spiral has the highest degree hub nodes, while the base the most peripheral nodes. Nodes sizes are proportional to their connectional degree, with colors reflecting each region’s lesioned percentage. The strongest 0.1% of edges between nodes, which highlight pairs of nodes with consistently high number of streamlines interconnecting them, are shown for illustrative purposes. **(C)** Plot of the probability of lesion voxels (*y*-axis) vs. connectivity degree for structural connectome nodes (*x*-axis). The red line is a fitted logistic regression model. **(D)** Plot of the probability of lesion voxels (*y*-axis) vs. the degree of the functional co-activation network nodes (*x*-axis). Figure adapted from Crossley et al. (2014), with permission.

Converging evidence supports the central role of brain connectivity in neurodegenerative progression. Abnormal connectivity might not only be involved in the propagation of downstream effects, it might also support upstream pathologic causes (Pievani et al., 2014). This supports the strategic importance of understanding the role of brain connectivity in disease evolution. Notably, the finding of patterns of pathology that reflect known structural connectivity, suggests the active role of specific epicenter nodes during the disease processes (e.g., deregulated genes, cell assemblies, and/or gross regions). In social networks, the presence of individuals with a disproportionately large number of contacts (social hubs) accelerates considerably the spread of infectious disease (Newman, 2002; Lloyd-Smith et al., 2005; Leventhal et al., 2015). This hub-centric behavior could be also be a feature of intra-brain pathologic propagations, not only limited to neurodegeneration but also present in other disorders (e.g., schizophrenia, epilepsy, Asperger’s syndrome). A recent meta-analysis study of 26 different brain diseases showed that disease-specific structural lesions were mainly located on connectivity hub regions (see Figure 5). This hub vulnerability could be a consequence of the high topological centrality and biological cost of the hubs, that make them more sensitive to a diverse range of pathogenic processes (Crossley et al., 2014). In addition, we showed that brain regions with a higher degree of anatomical connectivity experience early Aβ arrival and larger periods of Aβ exposition (Figure 3B; Iturria-Medina et al., 2014), which, in addition to excessive connectivity-dependent neuronal activity (de Haan et al., 2012), explains the higher Aβ deposition levels found on functional hubs (Buckner et al., 2009).

In spite of its biological relevance, it is not totally clear yet how to quantify the role of dynamic connectivity in disease evolution. For instance, although MP propagation have been modeled and studied by means of diffusion networks (Raj et al., 2012) and epidemic-like spreading (Iturria-Medina et al., 2014), the predictive power of these models still needs further validation. Similarly, there are promising advances in the modeling and understanding of neuronal/synaptic spreading across structural networks (Sotero et al., 2007; Valdes-Sosa et al., 2009; Sanz-Leon et al., 2013, 2015; Messé et al., 2015), but these methodologies also require additional predictive validation. Increasing evidence supports that gender have a substantial impact on structural and functional brain connectivity (Gong et al., 2011), which have been suggested to explain specific gender-related cognitive differences (Ingalhalikar et al., 2014). Gender is also associated to the risk of develop specific neurodegenerative diseases. For example, women are more likely to develop AD than men (Farrer et al., 1997; Damoiseaux et al., 2012), whereas men present a significant higher risk to develop PD (Wooten et al., 2004). Thus, in order to reach a realistic operational model of dynamic brain organization during aging and degeneration, it is essential to clarify how gender-related differences, from genetic, molecular, structural and/or functional levels, might modulate the role of brain connectivity in disease development and progression. I addition, it is still unclear if a given connectivity change should be interpreted as the result of a pathologic induced alteration or as the outcome of a compensatory change. The current lack of multi-factorial trajectory analysis and particularly the absence of robust causal models of disease progression, make still unfeasible to discriminate between connectivity associated upstream and downstream effects. A decreased connection could be reflecting either a pathologic alteration or a counteracting compensation mechanism, whereas an increased connection could be responding either to a compensatory mechanism or to a pathologic spreading effect. Importantly, it is also unclear how our understanding of the role of connectivity in disease progression could be translated into the development of effective therapeutic strategies. As Zhang et al. (2013) point out, targeting highly-connected genes in deregulated gene networks may be effective in disrupting disease-related networks for the purpose of therapy, but that could be at the cost of unknown adverse effects. How to diminish the outcome of negative effects after possible therapeutic interventions is still the subject of much scientific debate, Computer simulation modeling could help considerably the exploration of the effects of intervention strategies. For example, Proctor et al. (2013) modeled DNA damage, p53/GSK3 regulation, Aβ and tau dynamics to predict the intervention effects of Aβ immunization. However, in order to extend the simulation analyses, we will need a deeper understanding of the genetic, protean, metabolic, vascular, functional and structural aberrant interactions associated with aging and neurodegeneration.

## Conflict of Interest Statement

The authors declare that the research was conducted in the absence of any commercial or financial relationships that could be construed as a potential conflict of interest.
